# Evaluation of clinical usefulness of a commercial multiplex PCR for diagnosing mycobacterial infections: a multicenter study

**DOI:** 10.1128/spectrum.00175-26

**Published:** 2026-07-13

**Authors:** Maria Cano-Fernandez, Laura Barrado, Harold Bermudez-Marvalc, Maria Cabrera-Pineda, Irene Diaz-De-La-Torre, Diego Domingo, Sara Hernandez, Jesus Garcia-Martinez, Paula Lopez-Roa, Cristina Loras-Gallego, Maria-Carmen Muñoz-Egea, Maria-Jesus Ruiz-Serrano, Maria Simon, Marta Tato, Carlos Toro, Jose F. Valverde-Canovas, Laura Viñuela-Gonzalez, Jaime Esteban, Llanos Salar-Vidal

**Affiliations:** 1 Department of Clinical Microbiology, IIS-Fundacion Jiménez Diaz 218187 , Madrid, Spain; 2 Department of Clinical Microbiology, Hospital Universitario de Mostoles 16863, Móstoles, Madrid, Spain; 3 Microbiology Department, Hospital Universitario Principe de Asturias 16269, Alcala de Henares, Madrid, Spain; 4 Department of Microbiology, H.U. Puerta de Hierro-Majadahonda 16370, Majadahonda, Madrid, Spain; 5 Department of Microbiology, Hospital Clinico San Carlos 16267, Madrid, Spain; 6 Department of Microbiology, Hospital Universitario de La Princesa 16517, Madrid, Spain; 7 Microbiology Laboratory, Hospital Severo Ochoa, Leganes, Madrid, Spain; 8 Microbiology, Hospital Universitario de Fuenlabrada 16495, Fuenlabrada, Madrid, Spain; 9 Department of Microbiology, Hospital Universitario 12 de Octubre 16473, Madrid, Spain; 10 Department of Microbiology, Hospital Universitario de Getafe 16503, Getafe, Spain; 11 CIBERINFEC-CIBER de Enfermedades Infecciosas 637284 , Madrid, Spain; 12 Department of Microbiology and Infectious Diseases, Hospital General Universitario Gregorio Marañon 16483, Madrid, Spain; 13 CIBERES-CIBER de Enfermedades Respiratorias 568067 , Madrid, Spain; 14 Department of Microbiology and Parasitology, Hospital Central de la Defensa Gomez Ulla 221908, Madrid, Spain; 15 Department of Microbiology, Hospital Universitario Ramon y Cajal 16507, Madrid, Spain; 16 Department of Microbiology, Hospital Universitario La Paz 16268, Madrid, Spain; 17 Laboratory of Microbiology, Hospital Universitario Fundacion Alcorcon, Alcorcon, Spain; 18 Clinical Laboratory, UR Salud. Paseo de Europa, San Sebastian de los Reyes, Madrid, Spain; Houston Methodist Hospital, Houston, Texas, USA

**Keywords:** real-time PCR, molecular diagnosis, *Mycobacterium tuberculosis*, non-tuberculous mycobacteria

## Abstract

Rapid and accurate differentiation between *Mycobacterium tuberculosis* complex (MTBC) and clinically relevant nontuberculous mycobacteria (NTM) is essential for appropriate patient management but remains challenging with conventional diagnostic methods. We evaluated the clinical performance of a commercial multiplex real-time PCR assay for the direct detection of MTBC, *Mycobacterium avium* complex (MAC), and *Mycobacterium abscessus* complex (MABSC) in respiratory specimens. In this prospective multicenter study, 845 respiratory samples from patients with suspected mycobacterial infection were collected across 15 microbiology laboratories in the Madrid region (Spain). PCR results were compared with mycobacterial culture as the reference standard and with acid-fast bacilli (AFB) staining. Overall, 301 samples (35.6%) were culture-positive for mycobacteria. The multiplex PCR detected mycobacteria in 245 specimens, identifying MTBC (*n* = 147), MAC (*n* = 77), and MABSC (*n* = 21). For MTBC, sensitivity and specificity were 90.3% and 98.5%, respectively, increasing to 96.3% sensitivity in AFB smear-positive specimens. Sensitivity for MAC was 61.0% overall and 76.9% in AFB-positive samples, while MABSC sensitivity was 72.0%; specificity exceeded 99% for all targets. Culture identified additional NTM species not included in the PCR panel, underscoring the continued importance of culture for comprehensive diagnosis. In conclusion, this multiplex real-time PCR assay provides rapid and highly specific detection of MTBC and major NTM complexes directly from respiratory samples, with high sensitivity in smear-positive specimens. Its use may facilitate earlier differentiation between tuberculosis and NTM disease, supporting timely and appropriate clinical management.

**IMPORTANCE:**

This prospective multicenter study evaluates a commercial multiplex real-time PCR assay for the direct detection of *Mycobacterium tuberculosis* complex and important nontuberculous mycobacteria in respiratory samples. The inclusion of a large number of specimens (*n* = 845) collected across multiple hospitals reflects real-world diagnostic practice and strengthens the clinical relevance of the findings, which demonstrate high specificity and excellent sensitivity for tuberculosis, particularly in smear-positive specimens, supporting the value of rapid molecular diagnostics for timely patient management.

## INTRODUCTION

The genus *Mycobacterium* comprises species of major clinical and epidemiological relevance, broadly classified into the *Mycobacterium tuberculosis* complex (MTBC) and non-tuberculous mycobacteria (NTM) (1). Both groups are capable of causing chronic pulmonary disease as well as extrapulmonary and disseminated infections, particularly in individuals with underlying lung disease or immunosuppression. While tuberculosis remains one of the leading causes of infectious mortality globally, nontuberculous mycobacterial (NTM) pulmonary disease has emerged as an increasing public health concern, particularly in high-income settings. This rise is associated with aging populations, a higher prevalence of chronic lung diseases, and an increasing number of immunocompromised patients, including those with HIV infection, malignancies, or receiving immunosuppressive therapies. In several regions, the incidence of NTM infections now exceeds that of tuberculosis, highlighting their growing clinical and epidemiological relevance. Both MTBC and NTM represent significant public health challenges due to their impact on morbidity, healthcare burden, and the need for prolonged and complex treatments (2). The *Mycobacterium avium* complex, *M. xenopi, M. kansasii,* and *M. abscessus* are among the most clinically significant NTM species (3).

The diagnosis of NTM pulmonary disease is challenging because its clinical and radiological manifestations frequently overlap with those of tuberculosis. Conventional diagnostic methods, including acid-fast bacilli (AFB) staining and culture, are limited by low sensitivity or long turnaround times (4). Microscopy cannot discriminate between MTBC and NTM, and culture-based confirmation may require several weeks. This delay can lead to suboptimal clinical management, as treatment strategies differ substantially between MTBC and NTM infections. While tuberculosis typically requires standardized treatment regimens with well-established first-line drugs, NTM infections often require prolonged, species-specific, and sometimes less predictable antimicrobial therapies. Thus, rapid and accurate differentiation between MTBC and NTM is essential for guiding appropriate clinical decision-making and improving patient outcomes. To overcome these limitations, PCR-based molecular techniques have emerged as crucial tools for the rapid diagnosis of mycobacterial infections (5). To date, and despite major advances in molecular diagnostics for MTBC, the development of assays capable of reliably detecting and differentiating NTM at the species level directly from respiratory specimens remains limited. Most commercially available molecular platforms either target MTBC exclusively or detect NTM only at the group level, which has limited clinical utility given the substantial heterogeneity in virulence, disease manifestations, and antimicrobial susceptibility among NTM species.

The introduction of commercial multiplex real-time PCR platforms capable of simultaneously detecting multiple mycobacterial species represents a significant advancement in the syndromic diagnosis of mycobacterial infections. Current molecular tools, such as GeneXpert MTB/RIF and other nucleic acid amplification tests, allow rapid detection of MTBC and, in some cases, drug resistance, but are generally limited in identifying nontuberculous mycobacteria (NTM). In contrast, genotyping and sequencing methods provide detailed species identification, although they are more time-consuming, often require prior culture, and are less suitable for routine use. Multiplex real-time PCR assays offer the advantage of simultaneously detecting MTBC and clinically relevant NTM directly from clinical samples in a single reaction, enabling faster diagnostic results. In this context, the present study evaluates the clinical performance of the *Mycobacterium* Real-Time PCR kit (Vircell, Granada, Spain) for direct detection from respiratory samples in different hospitals in the Madrid area, Spain.

## MATERIALS AND METHODS

### Study design and sample collection

A prospective multicenter study was conducted from June 2024 to July 2025 in 15 clinical microbiology laboratories processing mycobacterial cultures from 24 hospitals in the Madrid area. Respiratory samples from patients with high suspicion of mycobacterial infection were included. Samples were processed according to each laboratory’s standard procedures, including AFB staining and decontamination of the samples prior to inoculation onto liquid and/or solid medium (6). Culture-positive samples were further identified to species or complex level using routine methods available at each participating laboratory (e.g., MALDI-TOF, line probe assays, or sequencing where applicable).

### Molecular detection assay

Molecular detection of mycobacteria was performed using the Mycobacterium Real-Time PCR kit (Vircell, Granada, Spain). This multiplex real-time PCR assay is designed for simultaneous identification of clinically relevant mycobacterial groups in human respiratory samples: *Mycobacterium tuberculosis* complex (MTBC), *Mycobacterium avium* complex (MAC), and *Mycobacterium abscessus* complex (MABSC). Within the *M. avium* complex, the test considers *M. avium* subsp. *avium*, *M. avium* subsp. *hominissuis*, *M. avium* subsp. *paratuberculosis,* and *M. avium* subsp. *silvaticum*, *Mycobacterium intracellulare*, and *Mycobacterium chimera*. The assay targets the IS1081 insertion sequence (MTBC, Texas Red/ROX channel), internal transcribed spacer regions (MAC, Cy5 channel; MABSC, HEX/VIC channel), and 16S rRNA gene (genus *Mycobacterium*, FAM channel), with human *RNase P* as internal control (Q705/Cy5.5 channel). Primer and probe sequences were not disclosed by the manufacturer.

The assay was carried out following the manufacturer’s instructions under aseptic conditions. The PCR results were not reported to the clinicians and did not influence patient management.

Briefly, approximately 1 mL of each sample underwent thermal inactivation at 95°C for 30 min. Nucleic acid extraction and PCR setup were performed using the MagXtract 3200 automated system (Chroma ATE Inc., Taoyuan, Taiwan). A volume of 600 μL of inactivated sample was processed with the SE001 pre-dispensed extraction kit (Vircell, Granada, Spain), including proteinase K digestion and magnetic bead-based purification, yielding 80 μL of eluate in approximately 20 min per batch. Five microliters of extracted nucleic acid was automatically dispensed into lyophilized reaction tubes for PCR amplification on a CFX96 Touch system (Bio-Rad).

### Comparative analysis of diagnostic methods

Multiplex PCR results were compared with conventional culture, which was employed as the reference standard for mycobacterial infection diagnosis. Additionally, the PCR results were compared with acid-fast bacilli staining findings when they were available. Laboratories were additionally queried regarding the use of other molecular assays for MTBC detection.

### Statistical analysis

Collected data were analyzed using R statistical software. Sensitivity and specificity were calculated for the overall PCR assay as well as for each specific target detected.

## RESULTS

During the study period, a total of 845 respiratory samples from patients with suspected mycobacterial infection were included. The most frequent sample type was sputum (*n* = 594), followed by bronchial aspirates (*n* = 146), bronchoalveolar lavage samples (*n* = 98) and bronchial lavage specimens (*n* = 7). The median age of the patients included was 66 years, and 50.06% of them were female.

Of all specimens, 301 (35.6%) were culture-positive for mycobacteria belonging to MTBC (*n* = 156), MAC (*n* = 120), and MABSC (*n* = 25). Among these culture-positive specimens, 238 (79.1%) were also positive by acid-fast bacilli (AFB) staining (Table 1). In 29 cases, the AFB was positive, but no mycobacteria were recovered in culture (Table 1). In five cases, PCR yielded a positive result, while culture and AFB stain were negative (three positive results for MABSC and two positive results for MTBC). In total, the multiplex PCR assay detected mycobacterial complexes in 245 specimens (29%), identifying MTBC (*n* = 147), MAC (*n* = 77), and MABSC (*n* = 21).

**TABLE 1:** Correlation between culture-positive results with AFB smear, and multiplex PCR results by mycobacterial group^*a*^

Mycobacterial group and culture results	Total	AFB smear positive	AFB smear negative	Multiplex-PCR with AFB smear positive	Multiplex-PCR with AFB smear negative
MTBC	156	138	18	131	8
MAC	120	79	41	60	12
MABSC	25	21	4	15	3
Negative	488	29	459	7	5

^*a*^ Cultures positive for other *Mycobacterium* complexes not included in the panel, as well as contaminated cultures, were excluded.

The diagnostic performance of the multiplex PCR assay for each mycobacterial group is summarized in Table 2. For MTBC, the assay showed high sensitivity (90.26%) and specificity (98.53%), with sensitivity increasing to 96.32% in AFB smear-positive specimens. Sensitivity for MAC was lower overall (61.02%) but also increased considerably in AFB smear-positive cases (76.92%), while MABSC showed moderate sensitivity (72.00% overall; 71.43% in AFB smear-positive specimens). Specificity remained very high across all groups (>99%). Considering all mycobacterial complexes together, overall sensitivity was 89.66% and specificity 97.48%. High PPV and NPV values were observed for all mycobacterial complexes, reflecting the reliability of the multiplex PCR assay for confirming or excluding mycobacterial infection, although PPV was slightly lower for MABSC.

**TABLE 2:** Diagnostic performance of *Mycobacterium* real-time PCR assay for detection of mycobacteria compared with culture

Mycobacterial group	Sensitivity (95% CI)	Sensitivity in AFB smear-positive (95% CI)	Specificity (95% CI)	PPV (95% CI)	NPV (95% CI)
MTBC	90.26% (84.44–94.44)	96.32% (91.63–98.80)	98.53% (97.00–99.41)	95.20% (91.74–98.67)	96.55% (94.94–98.16)
MAC	61.02% (51.61–69.86)	76.92% (66.00–85.71)	99.37% (98.17–99.87)	94.74% (89.72–99.76)	91.01% (88.56–93.46)
MABSC	72.00% (50.61–87.93)	71.43% (47.82–88.71)	99.58% (98.49–99.95)	85.71% (70.75–99.99)	98.76% (97.77–99.74)
Total	89.66% (89.08–93.22)	89.36% (85.00–92.50)	97.48% (95.65–98.69)	94.24% (91.31–97.17)	87.18% (84.38–89.98)

Nineteen cultures were contaminated with bacteria or fungi, and were therefore excluded from the sensitivity and specificity analysis. In two of these cases, the PCR panel yielded positive results for MAC and MTBC, respectively. The sample positive for MAC also showed a positive acid-fast stain, while the sample positive for MTBC was obtained from a patient already undergoing treatment for tuberculosis.

Further analysis of the *M. tuberculosis* results revealed that among PCR-positive but culture-negative samples (*n* = 7), the AFB stain was positive in four cases, and one sample was obtained from a patient with a previous episode of tuberculosis. In four additional cases, molecular testing performed by the participating laboratories (e.g., GeneXpert MTB/RIF or equivalent assays) also detected *M. tuberculosis* complex, supporting the PCR findings obtained with the evaluated kit. Moreover, in 251 cases, an additional rapid molecular assay for *M. tuberculosis* detection was routinely performed in the participating laboratories, and comparison with the evaluated PCR showed 86.6% (107/124) concordance among positive results and 80.01% (201/250) overall agreement.

In addition to the MTBC results, discrepant findings were also observed for other mycobacterial complexes. In one sample, two different mycobacterium complexes were detected by PCR (MABSC and MAC), whereas culture yielded growth of *M. abscessus* only. Analysis of discrepant MAC results revealed two PCR-positive but culture-negative cases; both patients had a documented history of infection caused by *M. intracellulare*.

Culture identified 37 isolates corresponding to species not targeted by the PCR panel. The detected species were *M. gordonae* (*n* = 9), *M. fortuitum* (*n* = 6), *M. mucogenicum* (*n* = 6), *M. lentiflavum* (*n* = 5), *M. scrofulaceum* (*n* = 2), *M. celatum* (*n* = 1), *M. chelonae* (*n* = 1), *M. interjectum* (*n* = 1), *M. shottsii* (*n* = 1), *M. xenopi* (*n* = 1), and *Mycobacterium* spp. (*n* = 4). Only two of these samples were positive by AFB staining.

**Fig 1:**
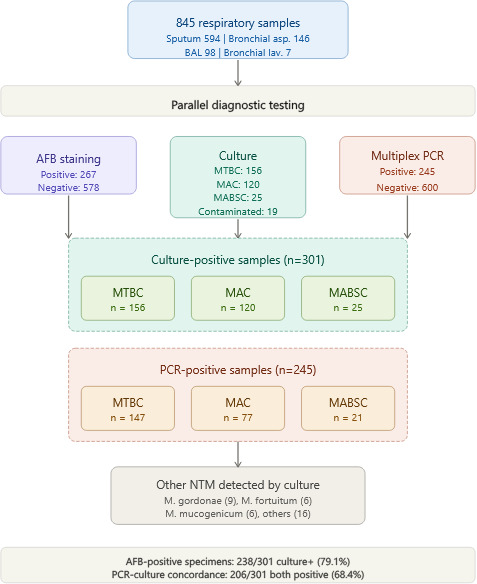
Flowchart of the samples included in the study.

A summary of the study workflow and the microbiological results obtained by the different diagnostic methods is shown in Fig. 1.

## DISCUSSION

Pulmonary NTM disease poses a diagnostic challenge due to its clinical and radiological overlap with tuberculosis. The increasing recovery of NTM species suggests a potential rise in mycobacteriosis (7–9). Although culture remains the diagnostic gold standard due to its high specificity, the slow growth of many mycobacterial species can delay definitive diagnosis for weeks or even months. This diagnostic delay negatively impacts clinical outcomes by postponing the initiation of appropriate therapy. In the case of NTM, delayed identification is particularly problematic, as these infections often require specific, prolonged, and species-directed antimicrobial regimens. In contrast, tuberculosis is usually treated with standardized first-line therapies, making early differentiation between MTBC and NTM essential for appropriate patient management. Advancements in molecular diagnostics and culture-independent techniques are crucial to detect and differentiate mycobacterial species in clinical practice, enabling earlier and more targeted treatment decisions. In this context, there is a critical need to improve diagnostic methodologies, which have traditionally focused on *M. tuberculosis*.

To overcome these limitations, PCR-based molecular techniques have emerged as important tools for the rapid diagnosis of mycobacterial infections. These molecular methods are increasingly valuable for the detection and species-level identification of NTM, which is essential given the diverse clinical relevance and varied antimicrobial susceptibility patterns of NTM species. Multiplex PCR from direct clinical specimens is a rapid and accurate method for the detection and differentiation of non-tuberculous mycobacteria from *M. tuberculosis* complex. These assays usually allow the simultaneous detection of MTBC and a range of clinically relevant NTM species directly from respiratory and extrapulmonary samples, with high specificity and variable sensitivity (10–16). The evaluated assay is performed on an open real-time PCR platform, increasing its flexibility compared with closed systems, such as GeneXpert, and offering a lower cost per sample in routine settings. Importantly, it enables direct identification of *Mycobacterium avium* complex and *Mycobacterium abscessus* complex from clinical samples, which is particularly relevant for clinical management and public health. In this multicenter study conducted across hospitals in Madrid, we evaluated the diagnostic performance of the new *Mycobacterium* real-time PCR (Vircell, Spain) in respiratory samples.

Our results confirm the high sensitivity and specificity of the assay for MTBC, especially in AFB smear-positive specimens, where sensitivity was above 96%. These findings are consistent with previous studies and support the use of multiplex PCR as a rapid and reliable tool for MTBC detection, with important clinical implications for timely treatment initiation and infection control (17–20). For NTM, the performance of the assay was lower, although the sensitivity increased considerably in AFB smear-positive specimens for MAC. This limitation has also been reported by other groups (11, 13) and likely reflects the heterogeneity and lower bacterial burden often associated with NTM pulmonary disease (21). Specificity remained very high for all NTM tested (>99%). The high PPV values (>90% for MTBC and MAC) indicate that a positive PCR result is highly predictive of culture positivity, whereas slightly lower NPV values suggest that negative PCR results should be interpreted cautiously, particularly in patients with low bacillary loads or clinical suspicion of NTM infection. These results suggest that the evaluated PCR assay is suitable for rapid detection of NTM and for distinguishing them from *M. tuberculosis* in clinical samples, which may facilitate timely and appropriate patient management.

Recent studies have shown that commercially available multiplex PCR assays for NTM differ in sensitivity, specificity, and the range of species detected. Most assays reliably differentiate MTBC from NTM, and some of them are also capable of identifying the major pathogenic NTM species. The spectrum of detection varies considerably, while certain assays are limited to 5–8 clinically relevant NTM, others cover up to 30 species. In general, these platforms demonstrated lower sensitivity for NTM detection than that for MTBC, although a high specificity across different platforms was observed (10–15). Our findings were consistent with these observations.

Furthermore, comparison of the evaluated PCR panel with other assays routinely used in the participating laboratories for MTBC detection showed a high degree of concordance, particularly among positive samples. Minor discrepancies between different methods may reflect differences in analytical sensitivity, target gene regions, or sample processing protocols. Notably, several PCR-positive but culture-negative results were also confirmed by those external molecular assays, supporting the reliability of the kit for MTBC detection.

Different discrepancies between PCR assay, culture, and AFB staining results were observed. Various samples were positive with the multiplex PCR assay (some of them also AFB smear-positive) and negative by culture obtained from patients with past mycobacterial infection, suggesting the persistence of mycobacterial DNA in the clinical specimens. Some acid-fast-positive specimens may yield negative culture results because conventional culture methods are primarily optimized for *Mycobacterium tuberculosis* complex isolation. The decontamination procedures used to prevent bacterial or fungal overgrowth rely on the relative resistance of *M. tuberculosis* to alkalis (22); however, non-tuberculous mycobacteria, particularly rapidly growing mycobacteria, are more susceptible to these reagents (22–25). As a result, viable NTM cells may be destroyed during processing, reducing their recovery in culture despite positive staining results. The simultaneous detection of MABSC and MAC may be explained by the rapid growth rate of MABSC, which can overgrow in culture and thereby mask the presence of more slowly growing mycobacteria such as MAC.

An additional challenge relates to the limited spectrum of species included in the evaluated panel. While the assay effectively identified the three targeted complexes, culture revealed a wider range of clinically relevant NTM species not detected by PCR. This highlights the continuing role of culture as an indispensable method for comprehensive identification and susceptibility testing, particularly in immunocompromised patients or in regions with diverse NTM epidemiology.

This study presents some limitations. Only pulmonary specimens were included, which limits extrapolation to extrapulmonary infections. In addition, although comparison with other molecular diagnostic methods was available in some cases, this was not systematically performed for all samples, as different molecular tools, including GeneXpert, were used according to routine clinical practice at each participating center. Although the multicenter design increases representativeness, all participating centers were located in Madrid, which may limit the applicability of the results to regions with different epidemiological patterns. Finally, lack of systematic clinical and radiological correlation limits assessment of the true clinical significance of discrepant results. Broader validation in extrapulmonary samples and in different epidemiological settings is warranted to fully establish the clinical value of multiplex PCR in the diagnosis of mycobacterial disease. In this regard, similar findings reported in a previous study (20), support the applicability of multiplex PCR in regions with different NTM epidemiology, reinforcing the generalizability and broader relevance of our results.

In conclusion, this new PCR kit for the detection of mycobacteria is a valuable tool in the molecular diagnosis of mycobacterial infections. It enables the direct identification of both tuberculous and non-tuberculous mycobacteria with excellent specificity across all species, and high sensitivity, especially when bacilloscopy is positive. These features highlight its usefulness as a rapid and reliable tool for diagnosing infections caused by these microorganisms, facilitating clinical decision-making and improving patient outcomes.

## Data Availability

Data of the study are available upon request.
